# Psychosocial correlates of fertility-related quality of life among infertile women with repeated implantation failure: The mediating role of resilience

**DOI:** 10.3389/fpsyt.2022.1019922

**Published:** 2022-11-11

**Authors:** Ying Ni, Limin Huang, Enming Zhang, Lianying Xu, Chenye Tong, Wen Qian, Aijun Zhang, Qiong Fang

**Affiliations:** ^1^Department of Gynecology and Obstetrics, Ruijin Hospital, Shanghai Jiao Tong University School of Medicine, Shanghai, China; ^2^Reproductive Medical Center, Ruijin Hospital, Shanghai Jiao Tong University School of Medicine, Shanghai, China; ^3^School of Nursing, Shanghai Jiao Tong University, Shanghai, China; ^4^Department of Nursing, Ruijin Hospital, Shanghai Jiao Tong University School of Medicine, Shanghai, China

**Keywords:** resilience, quality of life, psychosocial, repeated implantation failure, social support

## Abstract

**Objective:**

This study aimed to examine associations between psychosocial factors and fertility-related quality of life (FertiQoL) among infertile women with repeated implantation failure (RIF), and to identify the possible role of resilience in mediating the effect of social support on FertiQoL.

**Materials and methods:**

A cross-sectional study was conducted with 234 infertile women with RIF in total. Fertility quality of life scale (FertiQoL), perceived social support scale (PSSS), and Connor-Davidson Resilience Scale (CD-RISC) were used to evaluate the patients. Data were described by univariate and multivariate analyses. Stepwise regression method was performed to analyse the mediating effect of resilience.

**Results:**

Social support had a positive predictive effect on FertiQoL (β = 0.757, *P* < 0.001), also positive on resilience (β = 0.847, *P* < 0.001). After both variables were added to the regression equation, resilience was found to have a significant positive predictive effect on FertiQoL (β = 0.798, *P* < 0.001), while the predictive effect of social support on FertiQoL was no longer significant (β = 0.081, *P* > 0.05). The results indicated that resilience played a complete mediating role between social support and FertiQoL.

**Conclusion:**

This study preliminarily verified the mediating role of resilience between social support and FertiQoL among infertile women with RIF. Interventions that consider enhancing resilience and building social support will likely improve their FertiQoL.

## Introduction

With the delayed childbearing age, environmental pollution, life pressure, and other factors, the incidence of infertility is constantly increasing. Relevant data shows that the infertility is estimated to affect approximately 8–12% (1) of couples worldwide, 15.5% in China (2). With the development of *in vitro* fertilization and embryo transfer (IVF-ET) technology, more and more infertile patients realized their desire to have children. While the IVF treatment is successful in a considerable proportion of cases (3), there are still quite a few patients having experienced unsuccessful attempts, and approximately 25% having experienced more than five IVF cycles (4). Repeated implantation failure (RIF) is defined as the failure to achieve a clinical pregnancy after transfer of four or more high-quality embryos in a minimum of three fresh or frozen cycles in a woman under 40 years old (5). It is estimated that approximately 5–10% of women seeking IVF treatment will experience RIF (6).

Infertility, together with the treatment, is one of the greatest stressors in life (7), and leads to a variety of physical, psychological, and social consequences, which may significantly affect the fertility-related quality of life (QoL) of patients (8–10). QoL is defined by the World Health Organization (WHO) as an individual’s perception of his or her position in life within the cultural context and value system in which he or she lives (11). Accordingly, fertility QoL refers to the quality of life of individuals involved in emotion, body and mind, marriage, society, environment, and tolerance due to fertility problems (12), and reflects in a broad sense the living conditions of the infertile patients during the period of infertility. A large number of studies have shown that infertile women had a poorer QoL during infertility compared to the fertile counterparts (13–15). Compared with male spouses, women often undergo a large number of invasive surgery and monitor their menstrual cycle every day. In Chinese traditional culture, women bear the main pressure of infertility. Therefore, women will suffer more and have a significantly lower FertiQoL when facing the infertility crisis (16, 17). Repeated implantation failure aggravates the negative impact on infertile women.

Research by Coughlan et al. (18) investigated the psychological stress among infertile women, and found it significantly higher in stress level among women with RIF than those without RIF. After repeated failure of IVF attempts, RIF imposes a heavy financial burden and psychological distress on both patients and their families, and deeply affects their FertiQoL (19). The decline in QoL affects treatment compliance, which in turn affects pregnancy rates (20) and treatment outcomes (21).

Recent studies have shown that the main factors affecting the FertiQoL of infertile women include age, gender, education, marital relationship, duration of infertility, and emotional state (22, 23). Studies suggested that in addition to the general influence of clinical and demographic factors, psychosocial factors may also impact the FertiQoL of infertile women (24). Some researches have identified the psychosocial variables, including resilience and social support, that can alleviate the impact of infertility-related stress on FertiQoL among infertile women (25, 26).

Social support is an “available external resource” for individuals in the face of stress (27). It is usually defined as the perceived comfort, care, help and respect a person receives from others (28). Social support makes individuals believe that they are cared for and accepted, and at the same time, someone appreciates and takes care of them (29). It can help individuals reduce perceived stress, lessen impact of negative emotions, and improve quality of life. Recent studies revealed that social support was positively associated with FertiQoL (25, 30).

Resilience is a process of positive response and good adaptation when an individual is faced with traumatic event (31, 32). Patients with high level of resilience are typically perceived as having self-esteem, belief in their self-efficacy, and effective coping skills for stress (33, 34). Some studies also showed that resilience is positively correlated with FertiQoL in infertile women (35).

Research also shows that individuals with high perceived social support tend to have a high level of psychological resilience, which can enable individuals to adapt well to negative life events (36). In a cross-sectional study on American veterans, resilience was found to have a positive correlation with social support (37). Sippel et al. also revealed higher social support was related to greater resilience levels in trauma-exposed individuals (38).

However, there lack of studies specifically on RIF patients. Moreover, as far as we know, few published studies have explored the interrelationships between the three variables, and the mechanisms how social support and resilience synergistically influence FertiQoL among infertile women. This study aimed to examine associations between the psychosocial factors and FertiQoL among infertile women with RIF in China, and to identify the possible role of resilience in mediating the effect of social support on FertiQoL.

## Materials and methods

### Ethics statement

The study protocol was in accordance with ethical standards and was approved by the Ethics Committee of Ruijin Hospital. Written informed consent was obtained from each participant.

### Data and study design

An observational, cross-sectional study was conducted at the Reproductive Medical Center, Ruijin Hospital, Shanghai Jiao Tong University School of Medicine in China. All participants were recruited among women diagnosed with RIF, which was defined by Coughlan (5), and underwent IVF treatment from June to December 2021. The inclusion criteria were as follows: infertile women with RIF undergoing IVF, provided consent to take part in the study and had the ability to complete the survey. The exclusion criteria included women who were diagnosed with previous or current mental disorders, cognitive impairment, or severe chronic diseases.

After obtaining the written informed consent of this study, a self-reported questionnaire was distributed to each eligible participant, and clinical data were collected from their medical records. A total of 250 eligible participants were recruited, six patients declined to participate and 11 questionnaires were excluded for missing answers or the same answer for each question. In total, 234 complete responses were received with the effective rate of 93.6%.

*A priori* analysis using G*Power 3.1 was conducted to calculate the sample size required for this study (39, 40). The results showed that 107 was the minimum sample size needed to achieve sufficient power (95%) in detecting a medium effect size (f^2^ = 0.15). Thus, the number of participants (*n* = 234) in this study was an adequate sample size.

### Measures

#### Demographic characteristics

The demographic characteristics and clinical information were retrieved from medical records, including age, body mass index (BMI), residence, education, occupation, monthly income, types of infertility, attribution of infertility, number of IVF attempt cycles, and duration of infertility.

#### Measurement of fertility quality of life

The Chinese version of the fertility quality of life scale (FertiQoL) (12) was used to measure fertility-related QoL in this study. The scale is a self-assessment scale, including emotional, mind-body, marital, and social relations for the core module, tolerance and environment for treatment module, two independent items of subjective overall health status and overall QoL, with a total of 36 items. Likert 5-level scoring method is adopted for each item from 0 to 4 points. Higher score indicates the higher level of FertiQoL. The scale is widely used and has good reliability, validity and sensitivity (41, 42). In the present study, the Cronbach’s alpha coefficient of the FertiQoL Scale was 0.921.

#### Measurement of social support

The perceived social support scale (PSSS) translated and revised by Jiang Qianjin (43) was used. The scale emphasizes individual self-understanding and self-perceived social support, and measures the level of support perceived by individuals from family, friends and others respectively. The scale consists of 12 self-rating items, rated on a Likert 7-level scale ranging from 1 (Strongly disagree) to 7 (Strongly agree). Higher scores of each dimension and the overall level indicate higher level of social support. The scale is widely used in various fields and has been proven to have good reliability and validity (44). The Cronbach’s alpha coefficient of the PSSS was 0.941 in this study.

#### Measurement of resilience

The resilience was assessed by the Chinese version of Connor-Davidson Resilience Scale (CD-RISC) (45, 46). The questionnaire consists of three dimensions (tenacity, strength, and optimism), with a total of 25 items. A sum of higher total scores reflects greater resilience levels. The Chinese version of CD-RISC has shown sufficient reliability and validity (46, 47). In this study, Cronbach’s alpha coefficient of the CD-RISC was 0.937.

#### Statistical analysis

In this study, SPSS software version 23.0 was used for statistical analysis. The measurement data were expressed as the mean ± standard deviation, and the enumeration data were presented as the frequency and constituent ratio (%). Student’s *t*-test was used to compare the two groups. The x^2^ test was used to test the rate inspection. Pearson correlation test was used to perform the correlation analysis among social support, resilience, and FertiQoL in infertile women with RIF. Stepwise analysis was used for multivariate regression analysis on the predictors of FertiQoL and test the mediating effect of resilience on the relationship between social support and FertiQoL. All reported *P*-values were adjusted using the Benjamini-Hochberg (48) multiple comparison correction method (adjusted *P* < 0.05 for significance).

## Results

### Descriptive statistics

There were 76 (32.5%) patients with three failed IVF-ET cycles out of 234 participants of infertile women with repeated implantation failure, while 77 (32.9%) with four failed cycles, and 81 (34.6%) with five or more cycles. The average age of the 234 patients was 32.68 ± 3.63 years, ranging from 26 to 40 years old. The mean infertility duration and BMI was 5.38 ± 2.87 years and 21.72 ± 3.08 kg/m^2^, respectively. Characteristics of included participants are summarized in Table 1.

**TABLE 1:** Demographic characteristics of infertile women with repeated implantation failure (RIF).

Variables		Frequency	Percentage %
Age (years)	20–30	70	29.9
	31–35	104	44.4
	36–40	60	25.6
BMI (kg/m^2^)	<18.5	13	5.6
	18.5–23.9	186	79.5
	>23.9	35	15.0
Pregnancy history	Yes	105	44.9
	No	129	55.1
Duration of infertility (years)	≤3	67	28.6
	4–5	68	29.1
	≥6	99	42.3
Number of failed IVF cycles	3	76	32.5
	4	77	32.9
	≥5	81	34.6
Educational level	High school or below	74	31.6
	College/Bachelor	141	60.3
	Master or above	19	8.1
Residence	City	131	56.0
	Town	70	29.9
	Rural	33	14.1
Occupation	White-collar	153	65.4
	Blue-collar	41	17.5
	Unemployed	40	17.1
Monthly income (Yuan)	≤10,000	118	50.4
	10,001–15,000	60	25.6
	>15,000	56	23.9
Type of infertility	Primary infertility	127	54.3
	Secondary infertility	107	45.7
Attribution of infertility	Female factors	122	52.1
	Male factors	36	15.4
	Bilateral factors	76	32.5

The total FertiQoL score of RIF patients was 59.85 ± 11.51 points. The scores of the core module and the treatment module were 59.21 ± 13.72 and 61.37 ± 10.65, respectively. Among all the six dimensions, treatment environment got the highest score, while mind-body got the lowest. The total score of resilience was (62.71 ± 14.49). The tenacity dimension scored the highest, while the optimism dimension scored the lowest. The total score of perceived social support was (60.71 ± 11.69), which was in the medium level. Family support scored the highest, while friend support scored the lowest. Details are presented in Table 2.

**TABLE 2:** Fertility-related quality of life (FertiQoL), perceived social support scale (PSSS), and Connor-Davidson Resilience Scale (CD-RISC) scores.

Variables	Mean ± SD
Total score of FertiQoL	59.85 ± 11.51
Core module	59.21 ± 13.72
Emotional	55.38 ± 17.26
Mind-Body	53.86 ± 17.82
Relational	62.82 ± 12.95
Social	64.80 ± 17.99
Treatment module	61.37 ± 10.65
Environment	66.44 ± 11.80
Tolerability	53.77 ± 15.41
Total score of PSSS	60.71 ± 11.69
Family support	21.20 ± 5.06
Friend support	19.62 ± 4.60
Other support	19.89 ± 3.85
Total score of CD-RISC	62.71 ± 14.49
Tenacity	32.00 ± 7.74
Strength	21.29 ± 4.81
Optimism	9.42 ± 2.36

### Correlation between psychosocial variables and fertility-related quality of life

The Pearson correlation analysis showed that social support (PSSS) was positively correlated with FertiQoL (*r* = 0.757, Benjamini-Hochberg corrected *P* < 0.05), and resilience (CD-RISC) was also positively correlated with FertiQoL (*r* = 0.867, Benjamini-Hochberg corrected *P* < 0.05). Among them, the dimensions of family support and tenacity have the greatest correlation with FertiQoL, as shown in Table 3. It was indicated that the patients with higher resilience level of tenacity and more support from families would have higher FertiQoL. Moreover, there was also a positive correlation between social support and resilience (*r* = 0.847, Benjamini-Hochberg corrected *P* < 0.05).

**TABLE 3:** Correlation analysis between fertility-related quality of life (FertiQoL), perceived social support scale (PSSS), and Connor-Davidson Resilience Scale (CD-RISC).

Variables	1	2	3	4	5	6	7	8	9	10	11	12	13	14	15	16	17
1 Total score FertiQoL	1																
2 Core module	0.971**	1															
3 Emotional	0.851**	0.893**	1														
4 Mind-body	0.809**	0.848**	0.686**	1													
5 Relational	0.632**	0.589**	0.356**	0.287**	1												
6 Social	0.889**	0.932**	0.828**	0.731**	0.450**	1											
7 Treatment module	0.673**	0.477**	0.369**	0.354**	0.504**	0.387**	1										
8 Environment	0.440**	0.254**	0.105	0.100	0.511**	0.209**	0.832**	1									
9 Tolerability	0.657**	0.532**	0.517**	0.497**	0.285**	0.429**	0.771**	0.289**	1								
10 Total score PSSS	0.757**	0.755**	0.618**	0.568**	0.548**	0.755**	0.448**	0.379**	0.338**	1							
11 Family support	0.755**	0.738**	0.530**	0.660**	0.510**	0.724**	0.493**	0.456**	0.328**	0.842**	1						
12 Friend support	0.610**	0.642**	0.601**	0.463**	0.407**	0.630**	0.259**	0.184**	0.235**	0.894**	0.580**	1					
13 Other support	0.579**	0.558**	0.463**	0.304**	0.507**	0.591**	0.404**	0.332**	0.316**	0.865**	0.549**	0.758**	1				
14 Total score CD-RISC	0.867**	0.848**	0.728**	0.675**	0.562**	0.816**	0.564**	0.430**	0.479**	0.847**	0.738**	0.753**	0.703**	1			
15 Tenacity	0.853**	0.831**	0.718**	0.654**	0.548**	0.804**	0.569**	0.422**	0.497**	0.834**	0.728**	0.739**	0.693**	0.987**	1		
16 Strength	0.831**	0.818**	0.697**	0.660**	0.532**	0.791**	0.523**	0.431**	0.409**	0.792**	0.704**	0.697**	0.650**	0.963**	0.917**	1	
17 Optimism	0.831**	0.817**	0.698**	0.655**	0.572**	0.763**	0.529**	0.379**	0.478**	0.851**	0.709**	0.782**	0.721**	0.939**	0.914**	0.869**	1

**Indicates Benjamini-Hochberg adjusted *P* < 0.05.

### Regression analysis of the mediating role of resilience

**FIGURE 1:**
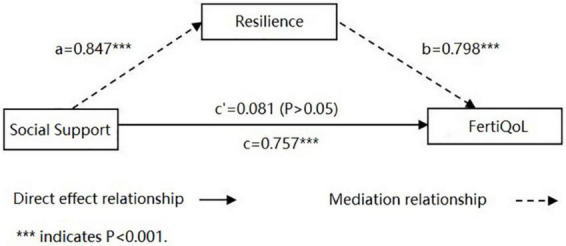
The model of mediation for the impact of social support toward fertility-related quality of life (FertiQoL) with the mediation impact of resilience. ***P < 0.001.

After controlling the demographic and clinical variables with statistically significant in the univariate analysis, including age, BMI, duration of infertility, etc., a stepwise regression method, designed by Wen et al. (49), was performed to further analyse the mediating effect of the three variables: FertiQoL as dependent variable (*y*), social support as independent variable (*x*), and resilience as mediating variable (*m*). In step 1, social support and FertiQoL were included into the regression equation, and it showed that social support had a positive predictive effect on FertiQoL (β = 0.757, *P* < 0.001). In step 2, social support and resilience were included into the regression equation, and the result showed that social support also had a positive predictive effect on resilience (β = 0.847, *P* < 0.001). In step 3, all the three variables were included into the regression equation. It showed that resilience had a significant positive predictive effect on FertiQoL (β = 0.798, *P* < 0.001). However, the predictive effect of social support on FertiQoL was no longer significant (β = 0.081, *P* > 0.05). Details are presented in Table 4. The results showed that social support had both direct and indirect effects on FertiQoL, and indicated that resilience played a complete mediating role between social support and FertiQoL (Figure 1).

**TABLE 4:** The regression results of the effects of perceived social support and resilience on fertility-related quality of life (FertiQoL).

Step	Outcome variable	Predictor	R	R2	F	β	*t*
1	FertiQoL	Social support	0.757	0.573	311.641***	0.757	17.653***
2	Resilience	Social support	0.847	0.717	588.546***	0.847	24.260***
3	FertiQoL	Social support	0.868	0.753	352.910***	0.081	1.319^n.s.^
		Resilience				0.798	12.992***

***Indicates *P* < 0.001, n.s. indicates *P* > 0.05.

## Discussion

Infertility, as an important crisis in marital life, increases the stress intensity of both couples (50). Repeated failed attempts have aggravated the physical and psychological trauma of the couples, especially women. However, this does not mean that all patients are desperate (51). There could be differences in perception of stress and ability to cope with difficulties among individuals. Some patients showed more confidence and optimism with sufficient supporting resources in effectively dealing with infertility (52). It is an effectual way to improve the FertiQoL of infertile women to use their own positive forces to fight against external negative factors. However, most studies mainly focused on the anxiety, depression and other adverse psychological status of infertile women, ignoring the positive psychological factors of individuals. Therefore, from the perspective of positive psychology, this study hopes to provide more insights on the mental health and FertiQoL of infertile women, especially those with RIF.

Resilience is a component of positive psychology. It not only means that an individual can be tough and tenacious under pressure and recover to the original state, but also emphasizes the growth and rebirth after trauma. This study showed that the resilience level of infertile women with repeated implantation failure was consist to previous studies (53, 54), and lower than the resilience of college students (55). Among all the three dimensions, the score of optimism was the lowest. This may be related to following factors. Firstly, infertile women have to endure various invasive injuries and adverse reactions of repeated IVF treatment. Secondly, Chinese traditional culture makes women bear great social pressure when facing infertility, which leads to the decrease of self-esteem. Moreover, the uncertain outcome and high cost of treatment also cause heavy burden on the family, which often leads to pessimistic feelings and experiences.

The results of this study showed that the mean score of perceived social support was 60.71, which was lower than that reported by Yu et al. (54). It may be related to the difference in the composition of the sample population. Among all the three dimensions, the score of family support was the highest. In Chinese traditional culture, people are accustomed to regard family and relatives as the main sources of support. On the other hand, the stigma brought by infertility makes patients reluctant to share it with people other than their families. They are more willing to seek support from family members when encountering difficulties.

The average FertiQoL score was 59.85 in this study, which was lower compared with the result among infertile women undergoing IVF from research by Karabulut (23). Ying et al. (56) reported that the FertiQoL of infertile women was significantly lower than that of women of childbearing age without infertility. Chinese traditional cultural concepts, social public opinion and heavy economic burden lead to the low FertiQoL of infertile women. Due to repeated attempt failures in the treatment process, RIF patients may suffer more physical pain, economic pressure, distress and disappointment, which results in lower FertiQoL.

This study also revealed that there were positive correlations among the resilience, social support and FertiQoL (Benjamini-Hochberg corrected *P* < 0.05). Previous studies have shown that social support is an important predictor of QoL (22, 54). People may need not only objective support, but also subjective support when facing stress, so as to make great support utilization. Perceived social support is the emotional experience and satisfaction of individuals who feel respected, supported, and understood. Queenen et al. (57) found that the social support perceived by cancer patients is more important than the quantity or degree of support. The higher the level of perceived social support, the easier it is for individuals to face adversity in a positive way, and the quality of life will be improved accordingly. Resilience is an important component of people’s mental health. It can help patients to deal with diseases with a brave, optimistic, and positive attitude. Therefore, the higher the level of resilience, the higher the QoL. As internal and external protective factors, resilience, and perceived social support both promote the improvement of individuals’ QoL.

The results of this study revealed that perceived social support and resilience have protective effects on the FertiQoL of infertile women with RIF. Further results indicated that social support can not only directly affect the FertiQoL, but also indirectly affect it through resilience. The direct impact comes from the material and emotional support from the family and society, which helps patients effectively integrate various resources when facing difficulties. The social support plays a buffer role, which enables individuals to adapt to difficulties and promotes the recovery of mental health (58). The mediating effect of resilience on social support and FertiQoL may be that patients with high resilience could perceive more support, which helps them overcome the adverse impact of disease, and more easily to take a positive and optimistic approach to deal with various emergencies effectively. There are higher levels of life satisfaction and happiness, in tune higher levels of FertiQoL.

## Limitations

There are some limitations in this study. First, self-reported measures may contribute to the likelihood for social desirability bias. Second, the convenience sampling in one center was adopted in this study, which might have affected the representation of the results to a certain extent. Third, this is a cross-sectional study. Further studies with a longitudinal design are needed to confirm the findings of this study.

## Conclusion

This study preliminarily verified the mediating role of resilience in social support and fertility-related QoL among infertile women with repeated implantation failure. The results showed that FertiQoL of infertile women with RIF improved with the increasing levels of social support and resilience. It is necessary to offer RIF patients more and sufficient respect, care, and support from family, friends and medical staffs. Interventions that consider enhancing resilience and building social support will likely improve their FertiQoL.

## Data availability statement

The raw data supporting the conclusions of this article will be made available by the authors, without undue reservation.

## Ethics statement

The studies involving human participants were reviewed and approved by Ethics Committee of Ruijin Hospital. The patients/participants provided their written informed consent to participate in this study.

## Author contributions

YN: conceptualization, methodology, writing – original draft and review and editing, and funding acquisition. LH: conceptualization, investigation, data curation, and writing – original draft. EZ: validation, formal analysis, and data curation. LX: conceptualization and project administration. CT: investigation, resources, and data curation. WQ: investigation, resources, and data curation. AZ: conceptualization and supervision. QF: conceptualization and supervision. All authors contributed to the article and approved the submitted version.
